# Contributions of viral oncogenes of HPV-18 and hypoxia to oxidative stress and genetic damage in human keratinocytes

**DOI:** 10.1038/s41598-023-44880-3

**Published:** 2023-10-18

**Authors:** Jimena Hochmann, Magdalena Millán, Paola Hernández, Laura Lafon-Hughes, Natali D’ Aiuto, Alejandro Silva, Juan Llaguno, Julia Alonso, Ariel Fernández, Vanesa Pereira-Prado, José Sotelo-Silveira, Ronell Bologna-Molina, Miguel Arocena

**Affiliations:** 1 Departamento de Genómica, Instituto de Investigaciones Biológicas Clemente Estable, Montevideo, Uruguay; 2 Departamento de Diagnóstico en Patología y Medicina Bucal, Facultad de Odontología, Universidad de la República, General Las Heras 1925, Montevideo, Uruguay; 3 Departamento de Genética, Instituto de Investigaciones Biológicas Clemente Estable, Montevideo, Uruguay; 4 Grupo de Biofisicoquímica, Departamento de Ciencias Biológicas, Centro Universitario Regional Litoral Norte -Sede Salto, Universidad de la República (CENUR LN, UdelaR), Montevideo, Uruguay; 5 Departamento de Biología Odontológica, Facultad de Odontología, Universidad de la República, General Las Heras 1925, Montevideo, Uruguay; 6 Instituto de Física, Facultad de Ingeniería, Universidad de la República, Montevideo, Uruguay; 7 Sección Biología Celular, Facultad de Ciencias, Universidad de la República, Montevideo, Uruguay

**Keywords:** Cancer, Cell biology

## Abstract

Infection with high-risk human papillomaviruses like HPV-16 and HPV-18 is highly associated with the development of cervical and other cancers. Malignant transformation requires viral oncoproteins E5, E6 and E7, which promote cell proliferation and increase DNA damage. Oxidative stress and hypoxia are also key factors in cervical malignant transformation. Increased levels of reactive species of oxygen (ROS) and nitrogen (RNS) are found in the hypoxic tumor microenvironment, promoting genetic instability and invasiveness. In this work, we studied the combined effect of E5, E6 and E7 and hypoxia in increasing oxidative stress and promoting DNA damage and nuclear architecture alterations. HaCaT cells containing HPV-18 viral oncogenes (HaCaT E5/E6/E7-18) showed higher ROS levels in normoxia and higher levels of RNS in hypoxia compared to HaCaT parental cells, as well as higher genetic damage in hypoxia as measured by γH2AX and comet assays. In hypoxia, HaCaT E5/E6/E7-18 increased its nuclear dry mass and both cell types displayed marked heterogeneity in nuclear dry mass distribution and increased nuclear foci. Our results show contributions of both viral oncogenes and hypoxia to oxidative stress, DNA damage and altered nuclear architecture, exemplifying how an altered microenvironment combines with oncogenic transformation to promote tumor progression.

## Introduction

Human papilloma viruses (HPVs) are non-enveloped small DNA viruses that infect keratinocytes of the skin and mucosa at different anatomic locations. The main risk factor for the development of cervical cancer and its precursor lesions worldwide is the persistent infection with a high-oncogenic risk HPV type (HR-HPV)^1^. HPV-16 and HPV-18 are associated with about 70% of all cervical cancers. HPV16 is the most frequent one, and it is associated with squamous cell carcinomas (SCC), while HPV18 is the second most frequent and often detected in adenocarcinoma^2^. The high-risk types of HPV encode three viral oncoproteins, E5, E6 and E7 that bind to and modulate numerous cellular proteins. These oncoproteins serve as the major initiators of cell transformation, interacting with cellular proteins controlling key aspects of cell proliferation. For instance, E5 interacts with members of the epidermal growth factor receptor family (EGFRs), causing an enhancement of ligand-dependent activation of the EGFR, and EGF-dependent proliferation of cultured human keratinocytes^3,4^. As for E6 from high risk HPVs, its main target is the tumor suppressor p53. E6 forms a ternary complex with E6-associated protein (E6AP) and p53 and induce the latter degradation by the ubiquitin-mediated proteolysis pathway^5,6^. E7 from high risk HPV binds and induces the degradation of the tumor suppressor protein retinoblastoma (pRb), an essential regulator cell cycle, which disrupts the interaction of pRb with E2F transcription factor, releasing E2F and promoting the transcription of genes involved in S phase induction^7^.

Multiple steps are involved in the progression from HPV infection to cellular transformation to cervical cancer. Among the main factors that promote malignant transformation are chronic inflammation and oxidative stress (OS). Chronic inflammation contributes to approximately 25% of human cancers^8^, and induces reactive oxygen species (ROS) and nitrogen species (RNS) in inflammatory and epithelial cells that can damage DNA leading to mutagenic lesions^9^. An increase in ROS production can potentially promote cervical carcinogenesis^10,11^. Nitric oxide (·NO) is another contributor to oxidative stress^12^. Previous studies have shown that women with cervical cytological changes caused by HPV infections exhibited increased cervical nitric oxide release^13^. Several studies have shown a link between hypoxia and increased oxidative stress^14,15^ and, on the other hand, oxidative stress has been linked to increased tumor genetic instability and invasiveness^16,17^. For instance, ROS and RNS can damage DNA to form mutagenic lesions, such as 8-oxo-7,8-dihydro-20 -deoxyguanosine (8-oxodG) and 8-nitroguanine^18,19^. On the other hand, DNA damage is a key event in HPV-induced carcinogenesis, which can lead to the development of genomic instability. Recently, it was observed that HR-HPV oncoproteins activate members of the DNA damage repair pathways, and this is critical for viral replication^20^. However, it has been shown that a persistent expression of E6/E7 from high risk HPVs in normal cells could result in DNA damage and chromosomal aberrations, and various mechanisms have been proposed to explain these observations, such as replication stress and centrosome amplifications^20,21^.

In this work, we studied the relationship between hypoxia, oxidative stress, and DNA damage in HaCaT E5/E6/E7 HPV-18 cells, which are human immortalized keratinocytes transduced with the three viral oncogenes (E5, E6 and E7) of Human Papilloma Virus type 18 (HPV-18), previously developed by our group and which constitute a cellular model of an intermediate stage in viral oncogenesis^22^. When cultured in an in vitro model of the hypoxic, tumoral microenvironment, which we have previously characterized^23,24^, HaCaT E5/E6/E7-18 cells markedly increase ROS and RNS production, and they simultaneously display increased DNA damage and altered nuclear architecture. Our results show that both hypoxia and oncogenic transformation contribute to increase oxidative stress and DNA damage, cooperating therefore in the path towards malignant transformation in HPV-induced carcinogenesis.

## Results

### ROS and RNS detection in normoxia and hypoxia

**Figure 1:**
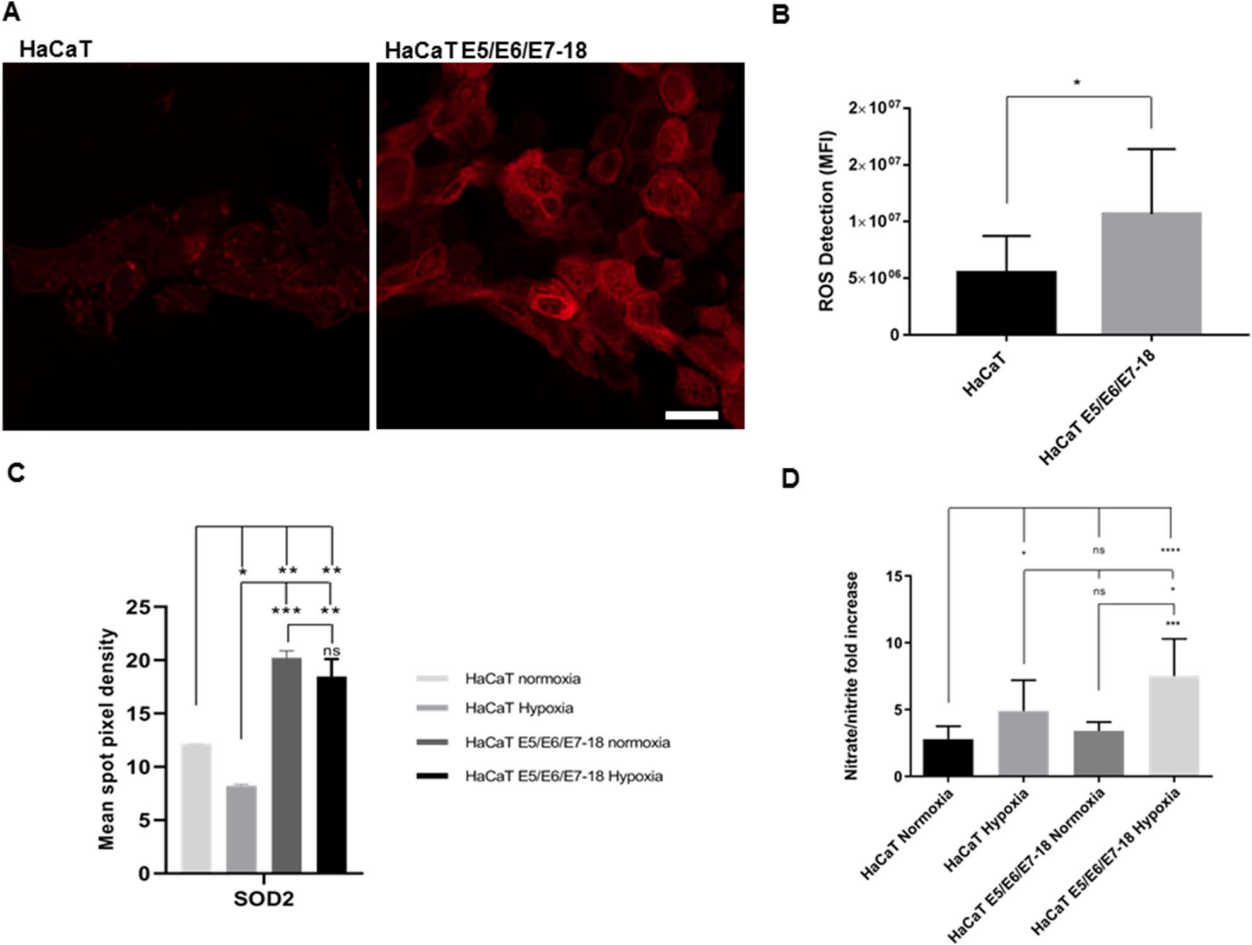
Detection of ROS and ·NO in HaCaT parental and HaCaT E5/E6/E7-18 cells. (**A**) Representative images of ROS Detection Reagent Deep Red signal. Scale bar: 20 μm. (**B**) Quantification of ROS intensity (mean ± SD) in normoxia for HaCaT parental and HaCaT E5/E6/E7-18 cells. Student`s Test was conducted to compare two means. (*) *p* < 0.05, (**) *p* < 0.01, (***) *p* < 0.001. (**C**) Relative expression levels of SOD2 expressed as mean spot pixel density is shown for HaCaT parental and HaCaT E5/E6/E7-18 cells as assessed by the Human Cell Stress Array Kit as suggested by the manufacturer (R&D Systems, MN, USA, ARY018). Averages ± standard deviations (SDs) of two independent experiments are shown. A two-way analysis of variance (ANOVA) test was conducted to assess statistical significance among groups along with Dunnett post-hoc test for intra-group comparison. (*) *p* < 0.05, (**) *p* < 0.01, (***) *p* < 0.001. (**D**) Nitrate/nitrite fold increase produced in HaCaT parental and HaCaT E5/E6/E7-18 cells in normoxia and coverslip induced hypoxia. Three independent experiments were conducted in triplicate. Mean ± SD is shown for each cell line. A one-way analysis of variance (ANOVA) test was conducted to assess statistical significance among groups along with Tukey’s HSD test for intra-group comparison. (*) *P* < 0.05, (**) *P* < 0.01, (***) *P* < 0.001, (****) *p* < 0.0001.

We evaluated the levels of intracellular ROS in HaCaT parental and HaCaT E5/E6/E7-18 live cells in normoxia by adding the ROS Detection Reagent Deep Red (640/675 nm excitation/emission) to cells and through visualization by confocal microscopy. We observed that cells containing viral oncogenes of HPV-18 (E5/E6/E7-18) exhibited significantly higher intracellular ROS compared to HaCaT parental cells (Fig. 1A,B). Furthermore, in agreement with our previous results, we detected higher expression levels of Superoxide Dismutase 2 (SOD2) in HaCaT E5/E6/E7-18 compared to HaCaT parental cells in both normoxia and hypoxia. In addition, it is interesting to point out that hypoxia seems not to alter SOD2 expression (Fig. 1C).

On the other hand, we also measured ·NO release by Griess method, and we observed that the production of ·NO in both HaCaT parental and E5/E6/E7-18 cells cultured under treatment with coverslips for 24 h was increased compared to cells cultured in normoxia. In addition, ·NO content was significantly higher in HaCaT E5/E6/E7-18 cultured in hypoxia compared to HaCaT parental cells (Fig. 1D).We also observed higher expression of Endothelial Nitric Oxide Synthase (eNOS) in HaCaT E5/E6/E7-18 cells cultures in normoxia and hypoxia compared to HaCaT parental cells, although it was not statistically significant (data not shown).

### DNA damage in normoxia and hypoxia

**Figure 2:**
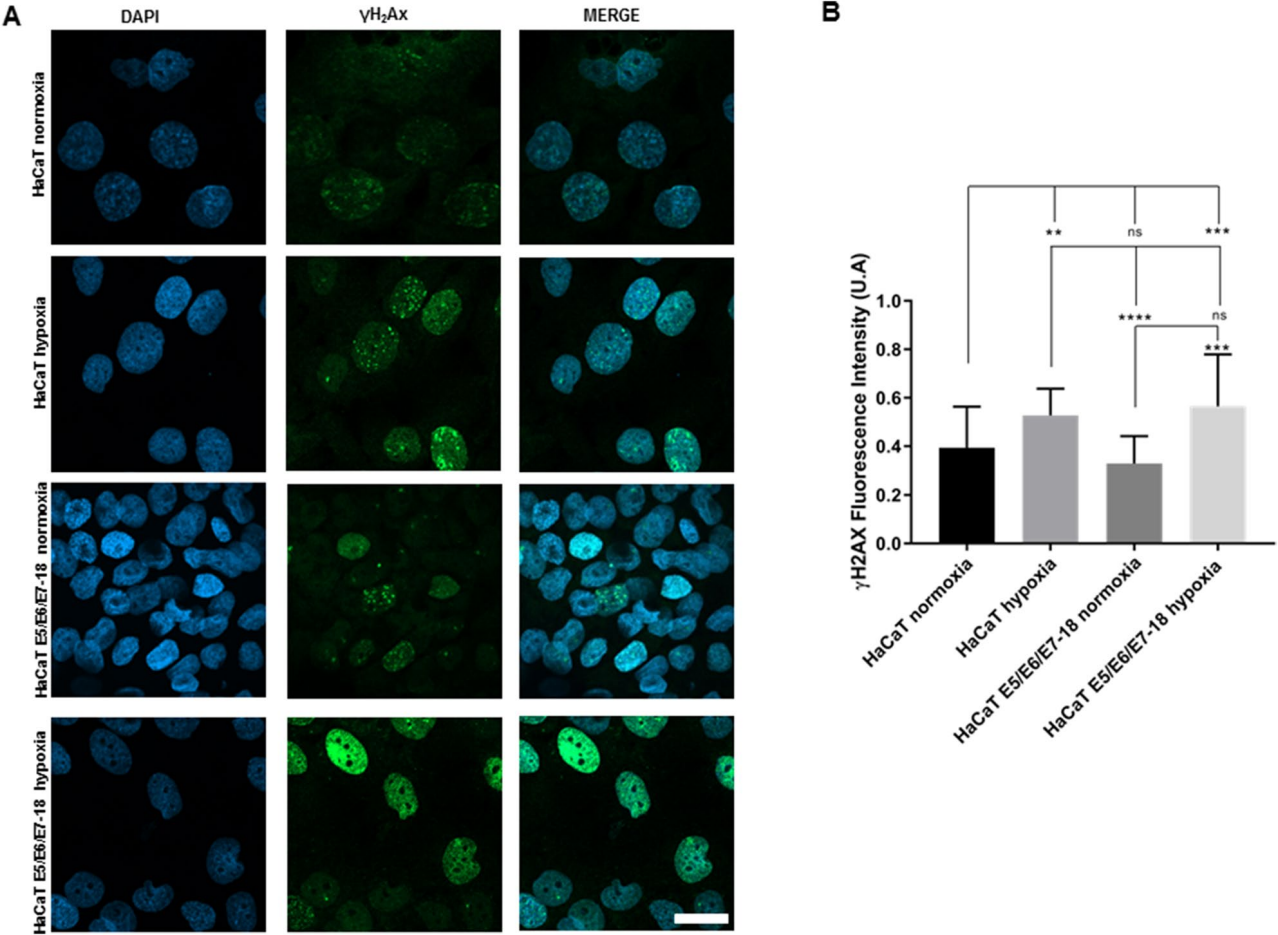
Detection of γH2AX signal by confocal microscopy. (**A**) Representative images of γH2AX signal in parental HaCaT and HaCaT E5/E6/E7-18 cells in normoxia and under treatment with coverslips for 24 h. Scale bar: 20 μm. (**B**) Quantification of γH2AX intensity (mean ± SD) in normoxia and coverslip induced hypoxia for HaCaT parental and HaCaT E5/E6/E7-18 cells. Three independent experiments were conducted. Mean ± SD is shown for each cell line. For all experiments, a one-way analysis of variance (ANOVA) test was conducted to assess statistical significance among groups along with Tukey’s HSD test for intra-group comparison (*) *P* < 0.05, (**) *P* < 0.01, (***) *P* < 0.001, (****) *p* < 0.0001.

**Figure 3:**
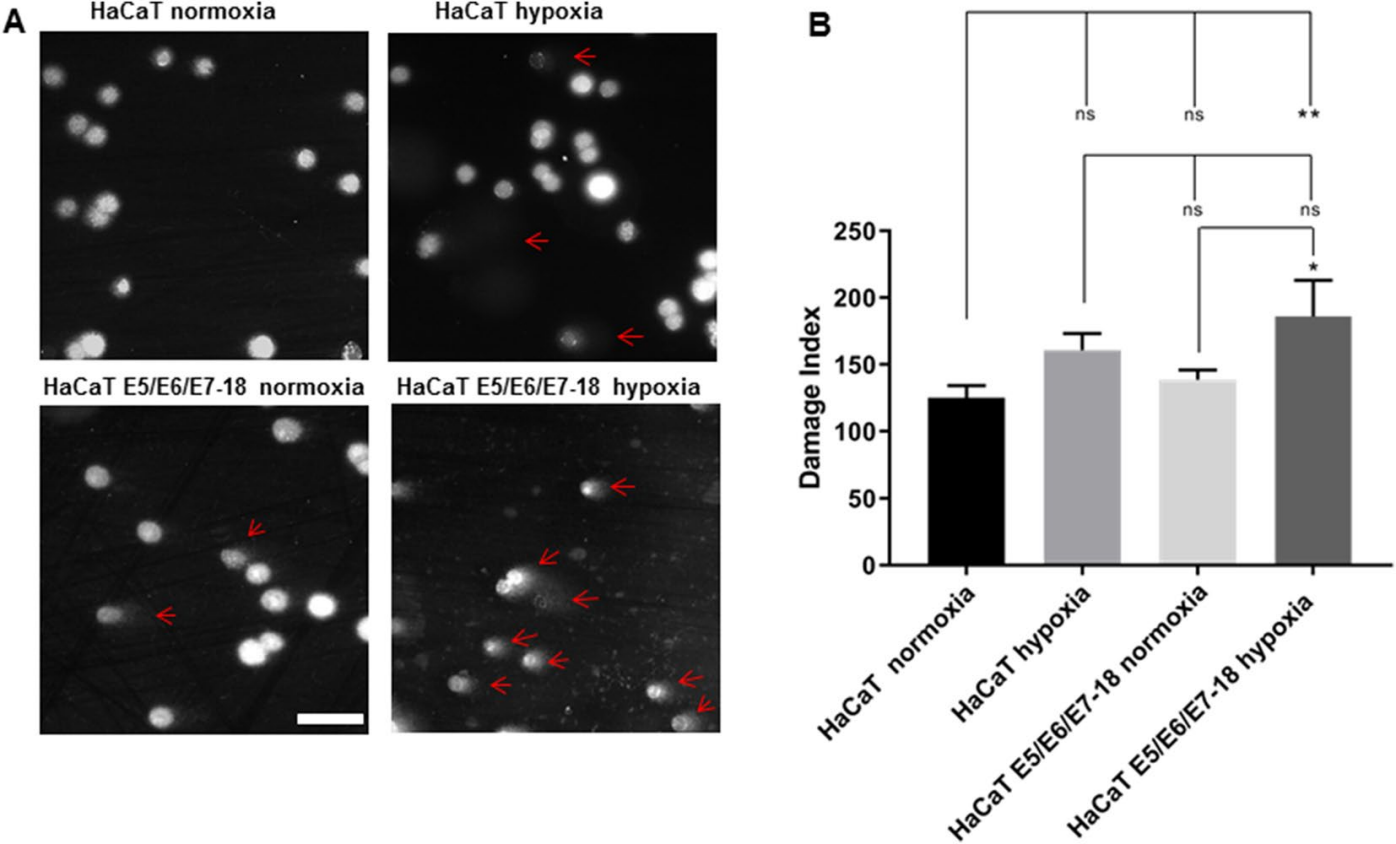
Comet assay in HaCaT parental and HaCaT E5/E6/E7-18 cell lines. (**A**) Representative comet images of HaCaT parental and HaCaT E5/E6/E7-18 cell lines stained with DAPI, in normoxia and in hypoxia visualized by epifluorescence microscopy following alkaline electrophoresis. The length of the comet tail is an indicator of the level of DNA damage (red arrows). (**B**) DNA damage index of HaCaT parental and HaCaT E5/E6/E7-18 cells measured in normoxia and coverslip-induced hypoxia. Two independent experiments were conducted in triplicate. Mean ± SD is shown for each cell line and condition. For all experiments, a one-way analysis of variance (ANOVA) test was conducted to assess statistical significance among groups along with Tukey’s HSD test for intra-group comparison. (*) *P* < 0.05, (**) *P* < 0.01, (***) *P* < 0.001.

Next, we assessed the contributions of both hypoxia and HPV-18 viral oncogenes in promoting DNA damage. First, we evaluated γH2AX levels in HaCaT parental and E5/E6/E7-18 cells both in normoxia and hypoxia. As shown in Fig. 2, both HaCaT parental and HaCaT E5/E6/E7-18 cells had significantly higher γH2AX fluorescence intensity in hypoxia compared to normoxia. However, no significant differences in γH2AX intensity were observed between HaCaT parental and HaCaT E5/E6/E7-18 cells either in normoxia or hypoxia, which suggests that hypoxia leads to greater γH2AX accumulation than viral oncogene expression. To further evaluate the level of endogenous DNA damage produced, we employed the alkaline comet assay (Fig. 3A). We observed a significant increase in the damage index in HaCaT E5/E6/E7-18 cells in hypoxia compared to normoxia, whereas the increase in the damage index in HaCaT parental cells in hypoxia compared to normoxia was not statistically significant (Fig. 3B). This suggests that hypoxia and viral oncogenes make a greater contribution to DNA strand breaks as measured by the comet assay than hypoxia alone.

### Nuclear dry mass density, dry mass and volume in normoxia and hypoxia

**Figure 4:**
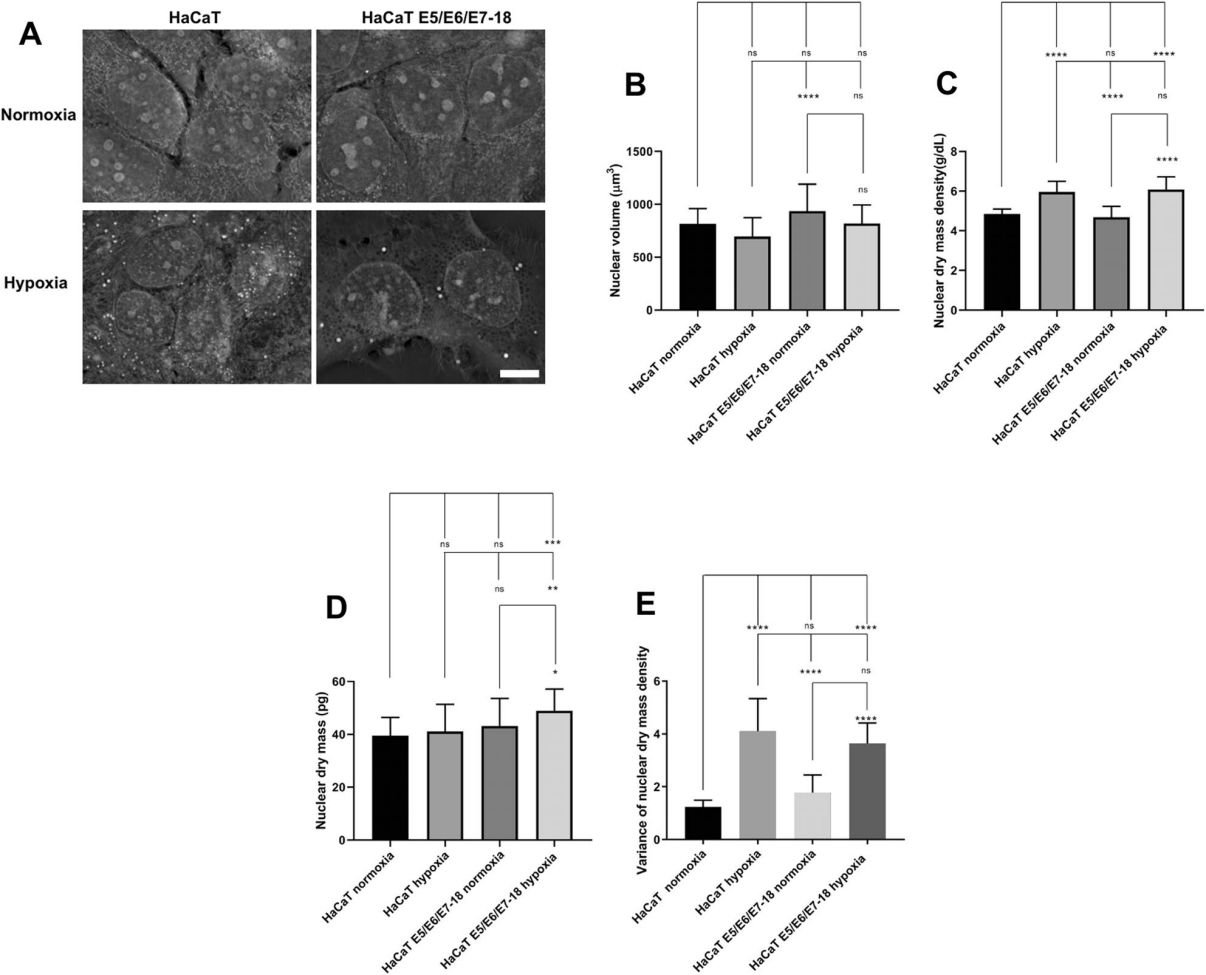
Quantitative phase imaging of HaCaT parental and HaCaT E5/E6/E7-18 cell lines. (**A**) Representative quantitative phase images of HaCaT parental and HaCaT E5/E6/E7-18 cell lines in normoxia and coverslip-induced hypoxia. Scale bar: 10 μm. (**B**) Nuclear volume in HaCaT parental and HaCaT E5/E6/E7-18 cell lines in normoxia and coverslip-induced hypoxia. (**C**) Nuclear dry mass density (g/dL) in HaCaT parental and HaCaT E5/E6/E7-18 cell lines in normoxia and coverslip-induced hypoxia. (**D**) Nuclear dry mass (picograms) in HaCaT parental and HaCaT E5/E6/E7-18 cell lines in normoxia and coverslip-induced hypoxia. (**E**) Variance of nuclear dry mass density in HaCaT parental and HaCaT E5/E6/E7-18 cell lines in normoxia and coverslip-induced hypoxia. For Figs. 4B, C, D and E, three independent experiments were conducted. Mean ± SD is shown for each cell line. For all experiments, a one-way analysis of variance (ANOVA) test was conducted to assess statistical significance among groups along with Tukey’s HSD test for intra-group comparison (*) *P* < 0.05, (**) *P* < 0.01, (***) *P* < 0.001, (****) *p* < 0.0001.

We then sought to analyze global changes in nuclear structure caused by hypoxia and viral oncogenes. To this end, we used the quantitative phase microscope Nanolive 3D Cell Explorer-Fluo, which allows to measure refractive index distributions inside cells, from which dry mass density distributions can be obtained, and which produces z-stacks from which cellular and subcellular volumes can be estimated. In hypoxia, for both cell lines, we observed marked changes in nuclei visualized by quantitative phase microscopy, with the appearance of brighter dots throughout the nuclear volume, suggesting that areas with increased dry mass density form inside nuclei in cells exposed to hypoxia (Fig. 4A and supplementary videos 1 and 2). We then obtained nuclear volume and nuclear dry mass density values, observing a small decrease in nuclear volume in hypoxia (Fig. 4B, not statistically significant), accompanied by a significant increase in nuclear mass density in hypoxia (Fig. 4C), suggesting that nuclei in hypoxia do not decrease their dry mass content even if they decrease in volume. Interestingly, nuclear dry mass content actually increases significantly in hypoxia for HaCaT E5/E6/E7-18 cells (Fig. 4D). However, the most marked change we observed was in nuclear dry mass density variance, which greatly increased in hypoxia for both HaCaT parental and HaCaT E5/E6/E7-18 cells (Fig. 4E), indicating a more heterogeneous dry mass distribution in nuclei in hypoxia, and suggesting that hypoxia results in marked changes in nuclear architecture.

### Nuclear foci quantitative image analysis

**Figure 5:**
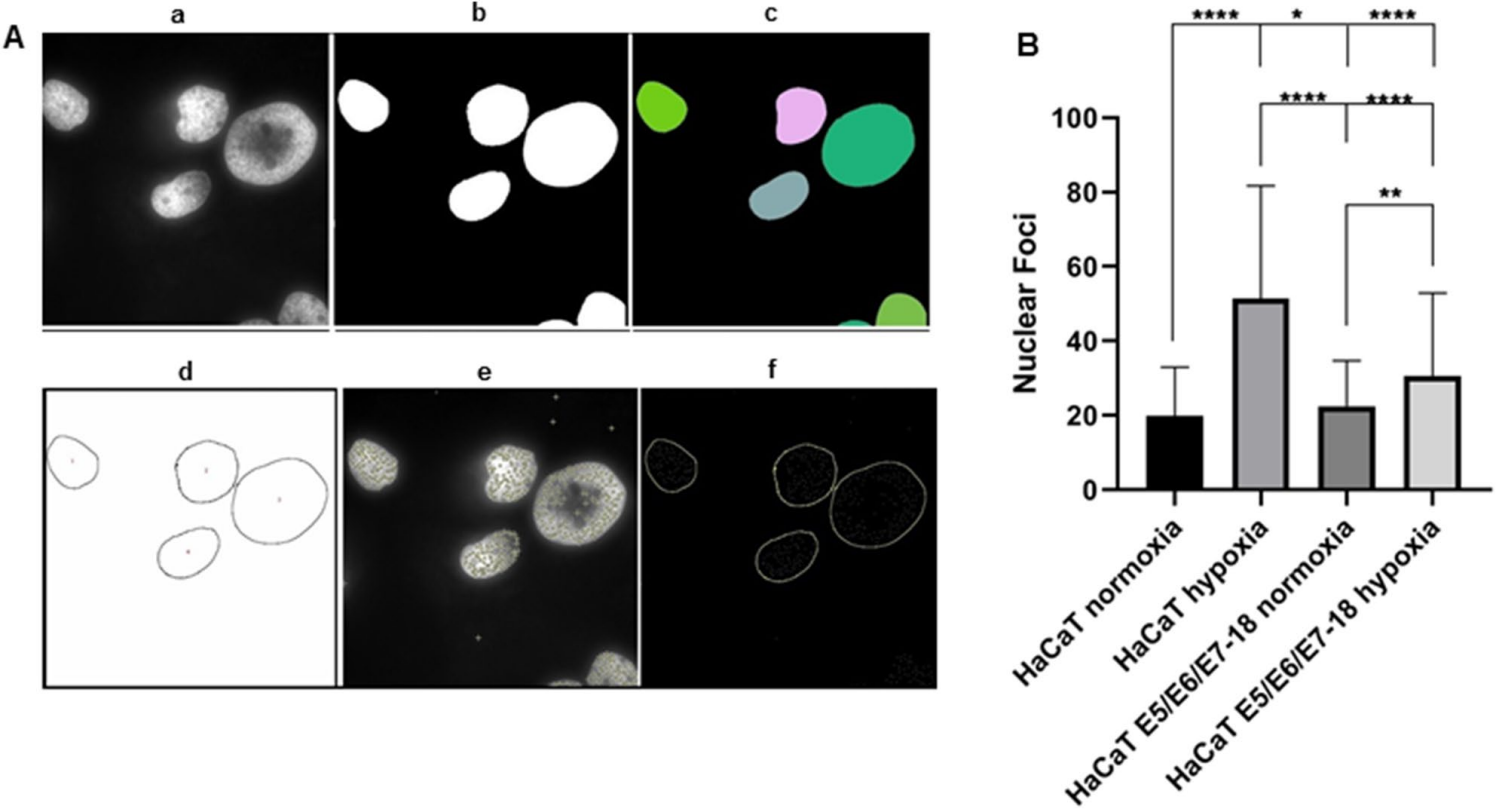
Nuclear foci quantitative image analysis (**A**) Segmentation of nuclear foci of a representative image of HaCaT E5/E6/E7-18 cells. a) Hoechst 33342 greyscale image. b) Threshold segmentation. c) StarDist 2D segmentation. Note that using this method the result is more precise than using only threshold, for example in the shape of the pink nucleus. d) Nuclear profile recognition and numeration. Note that on edge nucleus were excluded and only complete profiles were segmented. e) Find maxima single point tool for foci analysis. Isolated single points outside the nucleus were not considered. f) Combination of D and E for foci measurement. (**B**) Quantification of nuclear foci. For this analysis Mann–Whitney test and one-way analysis of variance ANOVA test was conducted to assess statistical significance among groups (*) *P* < 0.05, (**) *P* < 0.01, (***) *P* < 0.001, (****) *p* < 0.0001.

In hypoxia, the number of nuclear foci detected with Hoechst 33342 staining was significantly higher than in normoxia for both HaCaT parental and E5/E6/E7-18 cells, indicating an altered chromatin distribution after exposure to a hypoxic microenvironment, and confirming our previous results with quantitative phase imaging (Fig. 5).

## Discussion

In this work, we have studied the contributions of hypoxia and HPV-18 viral oncogenes to promoting oxidative stress and DNA damage, and therefore advancing the process of malignant transformation. HaCaT cells transduced with the E5, E6 and E7 HPV-18 oncogenes exhibited significantly higher intracellular ROS production compared to HaCaT parental cells. In addition, HaCaT E5/E6/E7-18 cells increased ·NO production in hypoxia and had significantly higher ·NO levels in this condition compared to HaCaT parental cells. Therefore, both HPV-18 oncogenes and hypoxia contribute to an increase in oxidative stress. Furthermore, hypoxia increased DNA strand breaks in both HaCaT parental and HaCaT E5/E6/E7-18 cells, while the extent of DNA damage was greatest in HaCaT E5/E6/E7-18 cells exposed to hypoxia, as measured by the comet assay, showing that HPV-18 oncogenes and hypoxia in combination contribute towards increased genotoxicity in addition to increased oxidative stress.

HPV oncogenes have been shown to cause increased oxidative stress levels, DNA damage and resistance to oxidative stress induced cell death: the E6 small isoform E6* increases oxidative stress and induces DNA damage^25^, whereas E7 modulates the expression of catalase, B-cell lymphoma extra-large (Bcl-xL), interleukin-8 (IL-8), Fas and Bad, resulting in resistance to oxidative stress-induced cell death in human keratinocyte cells^26^. More recently, it was reported that HPV-16 E6 and E7 induce oxidative stress and DNA damage in head and neck cancer cells^27^. We have also previously observed by flow cytometry that E5 in cooperation with E6 and E7 of HPV-18 promotes intracellular ROS production^22^, which we have now confirmed by confocal microscopy as well. In the present study, we have also observed increased expression in HaCaT E5/E6/E7-18 cells of SOD2. This enzyme catalyzes the dismutation of the superoxide (O^−2^) radical into ordinary molecular oxygen (O_2_) and hydrogen peroxide, it has key roles in controlling the redox status of tumor cells and is upregulated in several HPV-associated tumors, including penile and cervical carcinomas^28,29^, which is consistent with our results. We have also observed an increase, albeit not significant, in eNOS expression in HaCaT E5/E6/E7-18 cells, which is consistent with the increase in ·NO production by HaCaT E5/E6/E7-18 cells in hypoxia compared to HaCaT parental cells in the same condition. Increased levels of ·NO have been observed in women with cervical cytological changes likely related to HPV^13^, and this free radical can contribute to the induction of genotoxic lesions and to aspects of tumor progression such as cell growth, invasion and angiogenesis^30^, while also being an important component of the microenvironment in tumors such as cervical cancer, where it modifies immune responses and tumor behavior^31,32^. Therefore, the increase in ·NO produced by the combination of a hypoxic microenvironment and HPV oncogenes that we observed could be an important factor mediating the contribution of ·NO towards malignant transformation in HPV-related cancers, such as cervical cancers. More generally, our results suggest that the combination of microenvironmental hypoxia and HPV oncogenes can lead to increased oxidative stress during initial stages of tumor progression, likely contributing to advancing this process in HPV-associated cancers^33^.

HPV oncogenes induce both oxidative stress and DNA damage^20,21^ in different tumor types frequently associated with HPV infection, such as head and neck cancer^34^. Also, expression of HPV oncogenes E6 and E7 in normal cells results in DNA damage and genomic instability^21^, one of the hallmarks of HPV-induced carcinogenesis. Hypoxia has been associated with DNA damage as well, as for instance moderate hypoxia can cause indirectly DNA damage in the form of replication stress, as evidenced by S-phase arrest, ATR activation and phosphorylation of H2AX-Ser139 (γ-H2AX), p53-Ser15, Chk1- Ser345, and Chk2-Thr68^35,36^. In the present study, we evaluated DNA damage by assessing γH2AX nuclear accumulation and by the comet assay. γH2AX nuclear accumulation was markedly increased by hypoxia, but we did not observe differences between HaCaT E5/E6/E7-18 cells and HaCaT parental cells in normoxia. On the other hand, the DNA damage index measured by the alkaline comet assay increased in hypoxia for HaCaT E5/E6/E7-18 cells, but not for HaCaT parental cells. The γH2AX immunoassay is a sensitive technique to detect DNA double-strand breaks^37^, while the alkaline comet assay does not discriminate between alkali-labile sites and single or double-strand breaks^38,39^^.^ Therefore, these results suggest, firstly, that the hypoxia condition is the main responsible for inducing DNA double-strand breaks and that HPV-18 viral oncogenes alone had no significant implications in this type of DNA damage. On the other hand, the combination of HPV oncogenes and hypoxia increases the DNA damage detected by the alkaline comet assay in HaCaT E5/E6/E7-18 cells, which, taken together with the evidence of increased oxidative stress in hypoxia in these cells, suggests that hypoxia and HPV oncogenes combine to induce DNA damage consisting of single and double strand breaks and alkali-labile sites via higher oxidative damage.

Moreover, it is important to consider that early HPV infection produces DNA damage and upregulates DNA damage response genes, as shown for instance in a model of HPV infection using organotypic raft cultures of human cervical keratinocytes^40^. The combination of HPV infection and a hypoxic microenvironment could therefore result in increased selective pressure on the DNA damage repair capability of cells, leading to a cellular phenotype tolerant of higher DNA damage burdens and eventually to the emergence of resistant and aggressive tumors.

Genetic damage can cause profound alterations in nuclear architecture^41^. In this study, we used quantitative phase imaging to assess nuclear dry mass distribution as an indirect way to evaluate changes in nuclear architecture caused by hypoxia and HPV oncogenes. Our results indicate that nuclear dry mass density increases significantly with hypoxia, whereas nuclear volume tends to decrease, although not significantly. This would imply that the increase in nuclear dry mass density observed in hypoxia is mainly due to a decrease in nuclear volume and not an increase in nuclear dry mass. This appears to be the case for HaCaT parental cells, which do not show an increase in nuclear dry mass in hypoxia, whereas nuclear dry mass in HaCaT E5/E6/E7-18 cells increases in hypoxia compared to normoxia and compared to HaCaT parental cells in hypoxia.

A previous study has shown that nuclear dry mass is similar in SW480 and SW620 cell lines (derived from the same patient before and after metastatic spread), but that nuclear volume is smaller in metastatic SW620 cells^42^, suggesting that nuclear dry mass tends to be more conserved than nuclear volume. However, another study found that nuclear dry mass increases after ionizing radiation, in parallel with an increase in fluorescence intensity of γH2AX nuclear foci^43^, suggesting that an increase in nuclear dry mass might be related to the mobilization of DNA repair machinery. The increase in nuclear dry mass in HaCaT E5/E6/E7-18 cells in hypoxia might therefore be related to an increased DNA repair response, which could be caused in turn by the increased DNA damage observed in this condition using the comet assay.

We also observed a marked increase in nuclear dry mass density variance in hypoxia, indicating that the heterogeneity of the nuclear dry mass distribution increases in this condition, which implies that marked changes in nuclear architecture are taking place during hypoxia. Moreover, increased variance in dry mass distribution is linked to a decrease in cell stiffness^44^, whereas DNA damage has been shown to decrease nuclear stiffness^44^. Therefore, the marked increase in the variance of nuclear dry mass density that we observe in hypoxia would indicate that nuclear stiffness decreases in this condition, which could be related in turn to the increase in DNA damage observed. In summary, we have observed that both hypoxia and HPV oncogenes alter key physical nuclear parameters, which is likely related to the contributions of both factors to increased DNA damage. Moreover, as shown by Hoechst 33342 staining of live cells, hypoxia increases the number of nuclear foci, indicating chromatin redistribution in hypoxia, and therefore supporting the notion that hypoxia results in profound rearrangements of nuclear components. As future perspectives of this work, it will be important to identify if these foci contain components of the DNA damage repair machinery, and in particular which DNA damage repair pathways are activated in response to the combination of hypoxia and HPV oncogenes.

In conclusion, this is the first report that studies the contributions of hypoxia and the three viral oncogenes of HPV-18 to oxidative stress and DNA damage, and our results suggest that the interplay between microenvironmental hypoxia and HPV oncogenes is likely to cause increased oxidative stress and DNA damage, as well as an altered nuclear architecture, therefore propelling forward the process of HPV induced carcinogenesis.

## Materials and methods

### Cell lines

Spontaneously immortalized human keratinocyte (HaCaT) cells were purchased from Banco de Células do Rio de Janeiro (BCRJ), Brazil (batch number 001071, certificate of analysis provided by the supplier) and maintained in Dulbecco’s Modified Eagle’s Medium (DMEM) low glucose medium (Capricorn, Ebsdorfergrund, Germany) supplemented with 10% fetal bovine serum (FBS) (Gibco, Massachusetts, USA). HaCaT cells were transduced with three viral oncogenes (E5/E6/E7-18) of HPV-18 using lentiviral packaging systems. For this, HaCaT E5/E6/E7 HPV-18 cells were previously prepared by co-infection with a retroviral vector carrying the MSCV-N-puro-18E5 plasmid (Addgene # 37,882, MA, USA) and with a pLXSN retroviral vector that contained cloned both E6/E7 of HPV-18 genes that was kindly provided by Dra, Sichero from Instituto do Câncer do Estado de São Paulo. The preparation and characterization of these cell lines was detailed in Hochmann et al.^22^.

### Induction of hypoxia

In order to induce hypoxia to HaCaT parental and HaCaT E5/E6/E7-18 cells, we used the variant of coverslip-induced hypoxia method previously described ^23^. Briefly, this assay consists in culturing cells in the 10 mm radius wells of glass bottom dishes of 35 mm diameter (Cellvis, CA, USA) at increasing distances from an oxygen source. For this, after the cells were adhered to the wells, they were covered for 24 h with square acrylic coverslips of 24 mm of width and 2 mm of thickness with a square hole in the middle. In this way, an oxygen gradient is generated, with cells in the periphery of the chamber located farthest from the coverslip hole (the sole oxygen source).

### Intracellular ROS production

We evaluated the intracellular ROS production in live HaCaT parental and HaCaT E5/E6/E7-18 cells using ROS Detection Reagent Deep Red (Sigma, MO, USA, 1:1000 dilution from stock solution). Once cells reached a confluence of 60 per cent approximately, they were incubated with the reagent for one hour at 37 °C following manufacturer instructions. Then cells were visualized by confocal microscopy using a Zeiss LSM800 confocal microscope (Zeiss, Oberkochen, Germany) equipped with a Plan Apo N 63X oil NA 1.4 lens. Quantification of ROS intensity was evaluated using Image J software v. 1.43n4.

### Nitric oxide-releasing activity

In order to measure the ·NO production in our cells, we used the Griess reaction as we described previously^45^. This assay consists in measuring the nitrate/nitrite content in the cell culture medium. We performed this assay in normoxic and hypoxic conditions previously described. For the latter condition, cells were cultured under coverslips for 24 h, and after this period, cell supernatants were collected and analyzed. Briefly, 50 µl of culture medium were transferred to a new multiwell plate and incubated first with 1% sulfanilamide solution and then with 0.1% N-1-naphtilethylenrdiamine dihydrochloride protected from light. In parallel, a reference curve with NaNO_2_ was performed at serial dilutions between 0 and 100 µmol/l in 50 µl of culture medium. The absorbance was measured at 540 nm using a microplate spectrophotometer (Varioskan Flash Microplate spectrophotometer; Thermo Fisher, Vantaa, Finland).

### γH2AX immunocytochemistry

The DNA damage was evaluated by quantification of γH2AX nuclear immunofluorescence. For this, HaCaT parental and HaCaT E5/E6/E7-18 cells were seeded in a 24-well plate at density of 2 × 10^5^ per well and incubated for 24 h in a humidified 5% CO_2_ atmosphere at 37 °C. For the hypoxia condition, cells were cultured under coverslips for 24 h before the experiment. After that, culture medium was removed and cells were washed once with PBS. Then, cells were fixed for 15 min in 4% paraformaldehyde, rinsed in PBS three times, 5 min each, and permeabilized with blocking buffer (0.05% Triton X-100, 1% BSA in PBS) for 45 min at room temperature. Afterwards, permeabilized cells were incubated with the anti-γH2AX antibody diluted in blocking buffer (1/500, ab26350; Abcam, Cambridge, Massachusetts, USA) overnight at 4 °C. Finally, cells were washed with PBS three times for 5 min each, and incubated for 45 min with the secondary antibody (1:1000, goat anti-mouse conjugated with Alexa488; Invitrogen, Thermo Fisher Scientific, Waltham, Massachusetts, USA), and with DAPI (300 nmol/l; Invitrogen) to stain nuclei. Finally, cells were visualized using a Zeiss LSM800 confocal microscope. The γH2AX nuclear fluorescence was quantified related to DAPI signal for each condition, using the ImageJ software v. 1.43n4.

### DNA Damage measurement by comet assay

In order to evaluate the DNA damage generated in the hypoxic microenvironment in both HaCaT parental and HaCaTE5/E6/E7-18 cells, we used the alkaline comet assay. For this, HaCaT cells were plated in wells of 35 mm glass bottom dishes at densities of 2 × 10^5^ per well and incubated for 24 h in a humidified 5% CO_2_ atmosphere at 37 °C. For the hypoxia condition, cells were previously cultured under coverslips for 24 h. After that, cells were trypsinized and neutralized with culture medium. Then, cells were centrifuged for 5 min at 1200 rpm and the pellet was resuspended in 10 µl of PBS. The cell suspension (20 µl) mixed with 80 µl of 0.75% low melting point agarose (LMP) (Gibco #15,517–014) was extended on 1.0% NMP-agarose (Invitrogen #15,510–019) pre-coated slide. A coverslip was added and the agarose was allowed to set for 10 min at 4 °C. Then, the coverslip was removed and the cells were lysed by immersion of the slide in cold lysis buffer solution (pH 10, 2.5 mol/l NaCl, 100 mmol/l Na_2_EDTA, 10 mmol/l Tris–HCl, 1% Triton X-100) and 10% DMSO for 1 h at 4 °C. Electrophoresis was conducted in alkaline buffer (3 mol/l NaOH, 100 mmol/l EDTA, pH 13) at 25 V for 20 min in a cold unit at 4 °C with a distance between electrodes of 34 cms. Following neutralization, cells were stained with DAPI (6 µg/ml; Invitrogen) for 10 min. Two slides per condition were analyzed in each experiment and 100 cells per slide were scored. Damage was quantified as the damage index, which represents damage in individual cells DNA damage scores vary from 1 to 5, with 1 being the lower damage and 5 the higher damage. The Damage Index is calculated based on 100 cells, with the following equation: ${\sum }_{1}^{5}n.\alpha$ where n is the cell number and α is the damage score.

### Expression level of eNOS and SOD2

The relative intensities of eNOS and SOD2 proteins were evaluated using the Human XL Oncology Array Kit (R&D Systems, MN, USA, ARY026), and Human Cell Stress Array Kit (R&D Systems, MN, USA, ARY018) respectively following manufacturer instructions. Protein levels were quantified using the ImageQuant TL software (GE Healthcare, Buckinghamshire, UK), and the individual Western blot membranes were normalized according to the pixel densities of the 6 reference spots.

### Quantitative phase imaging and nuclear dry mass density calculation

HaCaT parental and E5/E6/E7-18 cells were imaged in normoxia or under thin glass coverslips to induce hypoxia using the quantitative phase microscope Nanolive 3D Cell Explorer-Fluo, which produces z-stacks of 96 slices with a depth of field of 30 μm. Cells were imaged for 24 h, and the refractive index distribution inside cell nuclei in normoxia and after 24 h under coverslips was obtained from the best focused slice, as each pixel in the quantitative phase images corresponds to a refractive index value. Next, refractive index values were converted to dry mass density values according to the equation^46^:

$$Drymassdensity\left( {g/dL} \right) = 665.\left( {\frac{RI}{{RI_{{H_{2} O}} }} - 1} \right)$$

where RI is refractive index and RI_H2O_ is the refractive index of water, equal to 1.33. Nuclei were segmented manually from quantitative phase images, and the average nuclear dry mass density and its variance were calculated using Python scripts. 30 nuclei were analyzed for each condition, and their volume was calculated from their areas in the slices in focus from the z-stacks. From the nuclear volume, and the nuclear dry mass density measured in the best focused slice, the estimated nuclear dry mass (expressed in picograms) was obtained.

### Nuclear foci quantitative image analysis

HaCaT parental and E5/E6/E7-18 live cells were stained with Hoechst 33342 (Sigma, MO, USA, final concentration 1 μg/ml) for 30 min either in normoxia or after 24 h under coverslips. Images were obtained with the fluorescence module of the Nanolive 3D Cell Explorer-Fluo and were analysed in order to determine the presence of nuclear foci. Normalization of the images was performed using Enhance Contrast tool of ImageJ, with 0,35% saturated pixels. Nuclear profiles were segmented with color threshold or ImageJ/Fiji StarDist 2D with the default parameters of the plugin^47^. Afterwards, overlapping or adjacent nuclei were separated with the macro Separate Labels^48^, and on edge nucleus were excluded. Nuclear foci were determined by Find Maxima ImageJ tool, which locates the maxima intensity in an image and, as a result, creates a binary mask of one segmented particle per maximum. Finally, both images were combined in order to measure the number of foci per nucleus.

### Statistical analysis

Statistical analysis and graphical presentation were performed using GraphPad Prism version 8.0.1 software (GraphPad Software Inc., San Diego, CA, USA). All experiments were performed in triplicate, and data were presented as the mean ± standard deviation (SD), with the exception of the comet assay, in which two experiments were performed induplicate. For all statistical analysis, One-Way unpaired ANOVA statistical test followed by Tukey’s HSD *post-hoc* test was conducted with the exception of data retrieved from ROS quantification, which was analyzed using Student`s Test in order to compare only two means, and data obtained from protein profile analysis which was analyzed using two-way analysis of variance (ANOVA) test in order to assess statistical significance among groups along with Dunnett post-hoc test for intra-group comparison. Statistical significance was determined at *p* < 0.05. Descriptive statistics, Mann–Whitney test and ANOVA was also performed for nuclear foci quantitative image analysis.

### Supplementary Information

Supplementary Information 1.

Supplementary Video 1.

Supplementary Video 2.

## Data Availability

All data associated with this study are present in the paper or the Supplementary Materials.

## Abbreviations

(HR-HPVs): High-Risk Human Papillomaviruses
(EGFR): Epidermal Growth Factor
(OS): Oxidative stress
(ROS): Reactive Oxygen Species
(·NO): Nitric oxide
(RNS): Reactive Nitrogen Species
(eNOS): Endothelial Nitric Oxide Synthase
(HIF-1/2): Hypoxia Inducible Factor 1/2
(SCC): Squamous cell carcinomas
(E6AP): E6-associatedprotein
(pRb): Protein retinoblastoma
(NOSs): Nitric oxide synthases
(8-oxodG): 8-Oxo-7,8-dihydro-20 -deoxyguanosine
(HaCaT): Spontaneously immortalized human keratinocyte
(BCRJ): Banco de células do Rio de Janeiro
(DMEM): Dulbecco’s Modified Eagle’s Medium
(FBS): Fetal bovine serum
(Bcl-xL): B-cell lymphoma extra-large
(IL-8): Interleukin-8
(BCRJ): Banco de Células do Rio de Janeiro
(DMEM): Dulbecco’s Modified Eagle’s Medium
(FBS): Fetal bovine serum
(Bcl-xL): B-cell lymphoma extra-large
(IL-8): Interleukin-8
