# Supplementary material for: Contributions of viral oncogenes of HPV-18 and hypoxia to oxidative stress and genetic damage in human keratinocytes
Source: Sci Rep. 2023 Oct 18;13:17734. doi: 10.1038/s41598-023-44880-3 (PMC10584980; doi:10.1038/s41598-023-44880-3)
Supplement: Supplementary file 1 — Supplementary Information 1. [file 41598_2023_44880_MOESM1_ESM.docx]

**Supplementary Video 1**. Time-lapse of HaCaT E5/E6/E7-18 cells cultured under coverslips for 24hr and imaged with quantitative phase microscopy. Images were taken at 10min intervals.

**Supplementary Video 2**. Time-lapse of HaCaT parental cells cultured under coverslips for 24hr and imaged with quantitative phase microscopy. Images were taken at 10min intervals.
